# Design, Preparation and In Vitro Evaluation of Core–Shell Fused Deposition Modelling 3D-Printed Verapamil Hydrochloride Pulsatile Tablets

**DOI:** 10.3390/pharmaceutics14020437

**Published:** 2022-02-17

**Authors:** Rui Li, Yue Pan, Di Chen, Xiangyu Xu, Guangrong Yan, Tianyuan Fan

**Affiliations:** 1 The State Key Laboratory of Natural and Biomimetic Drugs, School of Pharmaceutical Sciences, Peking University, Beijing 100191, China; 1410307125@pku.edu.cn (R.L.); 2111210046@stu.pku.edu.cn (Y.P.); 1310307112@pku.edu.cn (D.C.); 2 School Beijing Key Laboratory of Molecular Pharmaceutics and New Drug Delivery Systems, School of Pharmaceutical Sciences, Peking University, Beijing 100191, China; 3 School of Mechanical Engineering and Automation, Beihang University, Beijing 100191, China; xyxu1990@buaa.edu.cn (X.X.); yangr@buaa.edu.cn (G.Y.)

**Keywords:** FDM 3D printing, oral pulsatile tablets, verapamil hydrochloride, personalization

## Abstract

The aim of the study was to investigate core–shell pulsatile tablets by combining the advantages of FDM 3D printing and traditional pharmaceutical technology, which are suitable for a patient’s individual medication and chronopathology. The tablets were designed and prepared with the commercial verapamil hydrochloride tablets as core inside and the fused deposition modelling (FDM) 3D-printed shell outside. Filaments composed of hydroxypropylmethyl cellulose (HPMC) and polyethylenglycol (PEG) 400 were prepared by hot melt extrusion (HME) and used for fabrication of the shell. Seven types of printed shells were designed for the tablets by adjusting the filament composition, geometric structure and thickness of the shell. A series of evaluations were then performed on the 3D-printed core–shell tablets, including the morphology, weight, hardness, thermogravimetric analysis (TGA), differential scanning calorimetry (DSC), X-ray powder diffraction (XRD), in vitro drug release and CT imaging. The results showed that the tablets prepared by FDM 3D printing appeared intact without any defects. All the excipients of the tablet shells were thermally stable during the extruding and printing process. The weight, hardness and in vitro drug release of the tablets were affected by the filament composition, geometric structure and thickness of the shell. The pulsatile tablets achieved personalized lag time ranging from 4 h to 8 h in the drug release test in phosphate-buffered solution (pH 6.8). Therefore, the 3D-printed core–shell pulsatile tablets in this study presented good potential in personalized administration, thereby improving the therapeutic effects of the drug for circadian rhythm disease.

## 1. Introduction

Three-dimensional printing is defined as the “fabrication of objects through the deposition of a material using a print head, nozzle, or another printer technology” by the International Standard Organization (ISO) [1]. Since it was introduced in the 1980s, 3D printing has been widely used as a fast and economical technology in a variety of fields, such as aerospace, automobiles, architecture and jewelry. In medicine, 3D printing has been used in dentistry, tissue engineering and computer modeling of organs [2]. In the pharmaceutical field, 3D printing is still in its infancy [3] but has the potential to change the design and manufacture of medicines [4]. Three-dimensional printing has the characteristics of low cost, high efficiency and flexibility [5], and can support the design of medicines that have complex structures, can be produced on-demand and are tailored to the individual needs of each patient [6,7,8].

Fused deposition modelling (FDM) is currently the most widely studied 3D printing technology for solid preparation [9], providing good formability, resolution and mechanical properties [4]. In the process of FDM 3D printing, the polymer filament is melted in a high temperature nozzle and then extruded onto a platform layer by layer for deposition and solidification, forming 3D objects. Hot melt extrusion (HME) is now often used to prepare drug-loaded filament for FDM 3D printing preparation. Compared with the earlier method of producing drug-loaded filament by immersing blank filament in drug solution, HME can reduce the processing time and significantly improve drug content in filament, without the use of organic solvent [10,11]. FDM has been shown to offer a broad range of possibilities for the preparation of medicines [12]. However, the high temperature process of FDM 3D printing restricts thermal unstable drugs from being printed [13].

Recent studies have shown that many diseases have close relationships with circadian rhythms, such as heart disease, stroke, allergic rhinitis, asthma and arthritis and often attack in the early morning [14]. Based on these studies, a pulsatile drug delivery system has been developed to release drugs in a single or multiple dose at a predetermined time over several decades to prevent the diseases [15]. Recently, FDM 3D printing technology has been reported in the research of a pulsatile drug delivery system. To achieve delayed drug release, a printed caplet was coated with an enteric polymer [11], a dual- or multi-nozzle 3D printer was used to print a core–shell caplet or tablet with the drug-loaded part embedded in the drug-free part [16,17,18], and a powdery or liquid model drug was filled in the printed drug-free capsule [19,20,21,22].

In this study, verapamil hydrochloride was selected as a model drug. It is a calcium channel blocker used clinically to treat hypertension and angina [23], which typically occur when a patient is just waking up in the morning [24,25]. The core–shell pulsatile tablets were designed with the commercial verapamil hydrochloride tablets inside and the FDM 3D-printed shell composed of HPMC and PEG 400 outside. The design combined the advantages of traditional pharmaceutical technology in the aspect of good reproducibility, high safety and low cost, and of 3D printing technology in the way of flexibility, personality and on-demand, and meanwhile avoided the heat treatment of drug in the process of FDM 3D printing. Seven pulsatile tablets were designed and prepared using different filament compositions, geometric structures and thicknesses of the shells. A series of evaluations were performed to determine the factors effecting the quality of the tablets, and to confirm whether the 3D-printed core–shell pulsatile tablets could achieve the required release lag time and meet personalized administration.

## 2. Materials and Methods

### 2.1. Materials

Hydroxypropyl methylcellulose (Affinisol^TM^ HPMC HME 15lv) was kindly gifted by Dow (Miland, TX, USA). Polyethylene glycol (PEG 400) was purchased from Coolaber (Beijing, China). Commercial verapamil hydrochloride tablet was purchased from Shanghai Pharma Sine (Shanghai, China). Verapamil hydrochloride was purchased from Wuhan far Cheng (Hubei, China). Potassium dihydrogen phosphate (analytical grade) was purchased from Tong Guang Fine Chemicals (Beijing, China). Sodium hydroxide (analytical grade) was purchased from Beijing Chemical Industry Group (Beijing, China).

### 2.2. Preparation of Filaments

Three kinds of filaments were prepared by HME. Briefly, HPMC and PEG 400 were weighed accurately at a weight ratio of 90:10, 95:5 and 98:2, respectively, and blended evenly using a mortar and pestle [7]. Each mixture was extruded using a single-screw extruder (FilaBot^®^ FOV2 hot melt extruder, Filabot, Barre, VT, USA) equipped with a 1.75 mm nozzle at a working temperature of 140 °C. The filaments extruded were correspondingly named F90, F95 and F98, the diameter of which was controlled within the range of 1.65–1.75 mm [21].

### 2.3. Design and Preparation of Tablets

**Figure 1:**
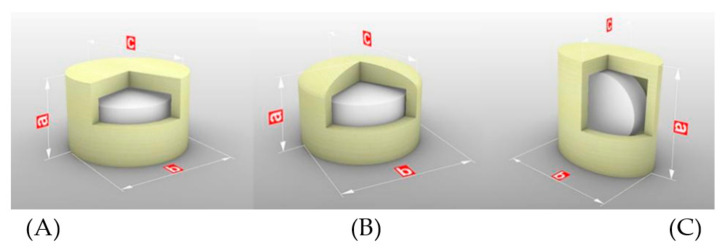
3D-printed tablets with different geometric structures: (**A**) FH-95-266, (**B**) CH-95-266, (**C**) FU-95-266. a, b and c represent the hight, diameter/major axis and minor axis of tablet, respectively.

**Figure 2:**
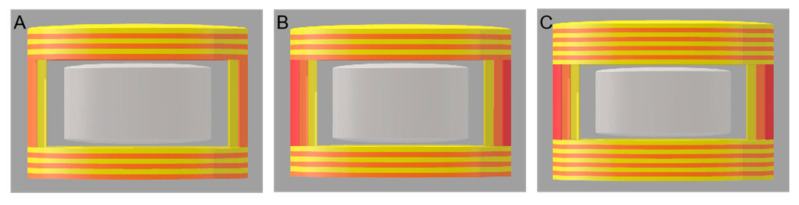
3D-printed tablets with different thicknesses of outer shell: (**A**) FH-95-266, (**B**) FH-95-366, (**C**) FH-95-399.

Core–shell pulsatile tablets were designed with the commercial verapamil hydrochloride tablets as core inside and 3D-printed shell composed of HPMC and PEG 400 outside. Seven kinds of 3D-printed tablets were designed using different filament compositions (F90, F95 and F98), geometric structures and thicknesses of shell (shown in Figure 1 and Figure 2 and Table 1).

The templates for printing the tablets were designed by 3D Builder 16.1.741.0 (Microsoft Corporation, Redmond, WA, USA) and exported as stereolithographic (.stl) files into the slicing software (Simplyfy3D 4.0.0, RepRap, Munich, Germany), which created the gcode file for the 3D printer. The shell of the pulsatile tablets was produced using a fused deposition modelling 3D printer (Mass Portal Pharaoh ED 3D printer, Mass Portal, Riga, Latvia) with the filaments prepared above. A brief pause (1–2 s) was made to place the commercial tablets inside the shell during the printing process. The printing parameters were set according to the thermal and mechanical properties of the filaments. For F90 and F95, the extrusion temperature and platform temperature were set at 200 °C and 100 °C, respectively. For F98, the extrusion temperature was set at 220 °C, and the platform temperature was set at 120 °C. For all filaments, the speed of extruder was set at 10–15 mm/s while extruding and 80 mm/s while traveling [26,27,28].

### 2.4. Morphology of Filaments and Tablets

Photographs of the filaments and tablets were taken with a Canon IXUS 220 HS digital camera (Canon, Tokyo, Japan).

Scanning electron microscopy (SEM) was used to observe the morphology of the surface and cross section of the filaments and tablets by a field emission scanning electron microscope (S4800, Hitachi, Tokyo, Japan) at 1.0 kV [29].

### 2.5. Weight and Size of Tablets

The tablets were weighed accurately by a digital analytical balance (ME104, Mettler Toledo, Shanghai, China) and measured with electronic digital display vernier callipers (KBD Tools, Jiangsu, China). The average and variance were calculated, respectively (*n* = 5) [26,30].

### 2.6. Hardness of Tablets

**Figure 3:**
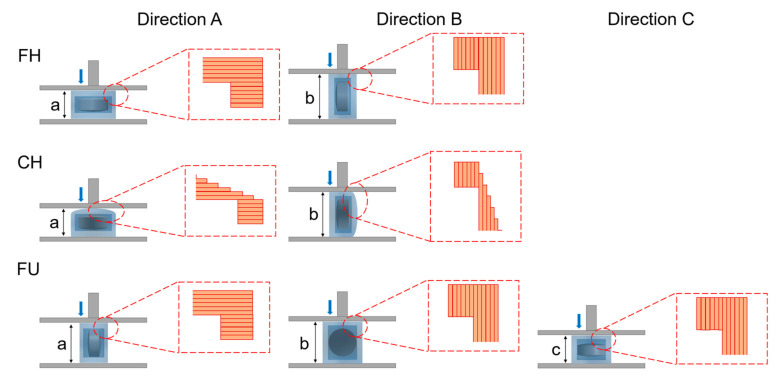
Illustration of the hardness testing in directions (**A**) force was added parallelly to the hight of tablet (a), (**B**) force was added parallelly to the diameter/major axis of tablet (b), and (**C**) force was added parallelly to the minor axis of tablet (c).

The hardness of the tablets was tested using a texture analyzer (MultiTest 2.5-i, Mecmesin, Horsham District, UK). A 75 mm probe with a speed of 1 mm/s and maximum force of 800 N was used to apply a vertical force to the tablet until it was fractured or deformed [31]. The hardness of the tablets was measured in two or three perpendicular directions, as shown in Figure 3 (*n* = 6).

### 2.7. Thermogravimetric Analysis

Thermogravimetric analysis (TGA) was performed by a thermal analyzer (Q600 SDT, TA Instruments, New Castle, DE, USA) to analyse the thermal decomposition of HPMC, PEG 400, physical mixture (HPMC and PEG 400), filament and the 3D-printed shell of the tablet. Samples (about 5 mg) were weighed accurately and placed in an aluminium crucible. The samples were heated from room temperature to 500 °C at a heating rate of 10 °C/min and a nitrogen gas purge of 100 mL/min. The data were analysed by an analysis software (TA 2000, TA Instruments, New Castle, DE, USA) [32].

### 2.8. Differential Scanning Calorimeter

Differential scanning calorimeter (DSC) was performed by a differential thermal scanning calorimeter (Q100, TA Instruments, USA) to analyse the thermal properties of the HPMC, physical mixture (HPMC and PEG 400), filament and 3D-printed shell of the tablet. Samples were weighed and placed in the aluminium crucible as the process in TGA assay. The samples were heated from −20 °C to 280 °C at a heating rate of 10 °C/min and a nitrogen gas purge of 50 mL/min. The data were analysed by an analysis software (TA 2000, TA Instruments, USA) [32].

### 2.9. X-ray Diffraction

X-ray powder diffraction (XRPD) was tested to assess the crystal structure of HPMC, physical mixture (HPMC and PEG 400), filament and 3D-printed shell of the tablet by an X-ray powder diffractometer (X’Pert Pro, PANalytical, Almelo, The Netherlands). Samples were scanned from 5° to 60° 2θ at a speed of 5°/min with a Cu X-ray source. The operating current and voltage were 15 mA and 40 kV [33].

### 2.10. In Vitro Drug Release of Tablets

The in vitro drug release of tablets referred to the United States Pharmacopeia (USP) dissolution apparatus I (RC-6 Intelligent dissolution tester, Tianjin New Tianguang, China) and the previous literature [34]. The tablets were placed in 900 mL of pH 6.8 phosphate-buffered solution (PBS, *n* = 3). The dissolution medium was maintained at 37 ± 0.5 °C and stirred at 100 rpm. During the test, 10 mL of samples were withdrawn at each predetermined time and were replaced immediately with an equal volume of fresh dissolution medium at the same temperature. The samples were filtered using an 0.2 μm filter, and the subsequent filtrates were taken and diluted appropriately. Drug concentration was determined by a UV spectrophotometer (UV-1100, Mapada, Shanghai, China) at 229 nm according to a standard curve pre-made between absorption and known drug concentration. The drug release curve was drawn, and the delayed release time was extrapolated.

### 2.11. Computed Tomography Imaging

The changes in pulsatile tablets during the dissolution process were observed by the representative tablets (FH-95-266). Six tablets were successively taken out at a predetermined time (0, 2.0, 3.0, 4, 4.5, 5.0 h) under the conditions of the dissolution test, followed by freeze-drying by a vacuum freeze dryer (FD-1B-80, Boyikang, Beijing, China). Photographs of the tablets were taken before and after freeze-drying. Computed tomography (CT) images of the dried tablets were conducted by a CT scanner (uCT 760, United imaging healthcare, Shanghai, China). The imaging parameters were as follows: thickness, 0.8 mm; 120 kVp, 266 mA; gantry rotation time, 0.5 s; and table speed, 158.9 mm/s [35].

### 2.12. Statistical Analysis

The data were analysed using t-test. Differences in results where *p* < 0.05 were considered significant, and *p* < 0.01 were considered extremely significant.

## 3. Results and Discussion

### 3.1. Preparation of Filaments

HPMC and PEG are two of the most widely used excipients in oral drug delivery systems [36,37]. HPMC was chosen as the hydrophilic matrix material and PEG 400 was adopted as the plasticizer in this study [22,28]. The use of plasticizer can lower the processing temperature of HME and adjust the thermoplasticity of the filaments [4,38,39]. The ratio of HPMC and PEG 400 was screened in the HME experiments of the prepared filaments with the appropriate mechanical properties and melt viscosity for successful printing. At a higher ratio of plasticizer, the obtained filaments were soft and difficult to feed into the 3D printer, and the printed tablets were not dense enough. At a lower ratio of plasticizer, the processing temperature was raised and the colour of the filaments tended to turn dark. Finally, the ratios of HPMC and PEG 400 were adopted at 90:10, 95:5 and 98:2 (*w*/*w*). All three kinds of filaments proved to be successful in printing the pulsatile tablets.

### 3.2. Design and Preparation of Tablets

Core-shell pulsatile tablets were designed with different filament compositions, geometric structures and thicknesses of shell to achieve personalized lag time in the release of the pulsatile tablets (Table 1).

HPMC is a sustained-release or controlled-release material, and PEG is a water-soluble substance. Different weight ratios of HPMC and PEG 400 in filaments (F90, F95 and F98) were first designed to make the difference in dissolution and destruction rate of the 3D-printed shell (FH-90-266, FH-95-266 and FH-98-266).

Considering that the cylindrical shape is one of the most popular shapes used for oral tablets [40], it was selected and modified. Three kinds of geometric structures were designed for the pulsatile tablets to investigate the effect of top structure/filling patten (FH-95-266 vs. CH-95-266) and top bottom surface area (FH-95-266 vs. FU-95-266) on drug release. The flat top and bottom of tablets (FH, flat top with core tablets placed horizontally) were filled with the grid format of orthogonal crossing, which allows the existence of holes. The tablets with convex top in the annular and dense filling pattern (CH, convex top with core tablets placed horizontally) were designed, and the tablets with small surface aera of top and bottom (FU, flat top with core tablets placed vertically) were designed as well.

The thickness of the shell was reported to affect its swelling, dissolution, erosion, water permeation and drug diffusion [39]. In order to achieve a suitable overall size for the pulsatile tablets for easy self-administration, 2 or 3 shells of side wall, 6 or 9 layers of top and 6 or 9 layers of bottom were designed.

### 3.3. Morphology of Filaments and Tablets

**Figure 4:**
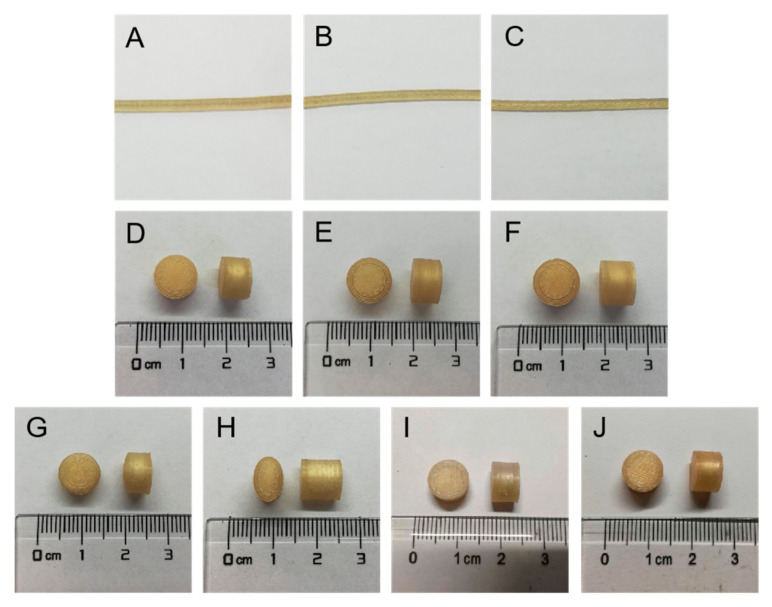
Photographs of the filaments and tablets: (**A**) F90, (**B**) F95, (**C**) F98, (**D**) FH-90-266, (**E**) FH-95-266, (**F**) FH-98-266, (**G**) CH-95-266, (**H**) FU-95-266, (**I**) FH-95-366, (**J**) FH-95-399.

The photographs of the filaments and tablets are shown in Figure 4. The surface of the filaments and tablets were smooth and free from bubbles and defects. No obvious difference was found between the three filaments. All the tablets showed the expected morphology as designed.

**Figure 5:**
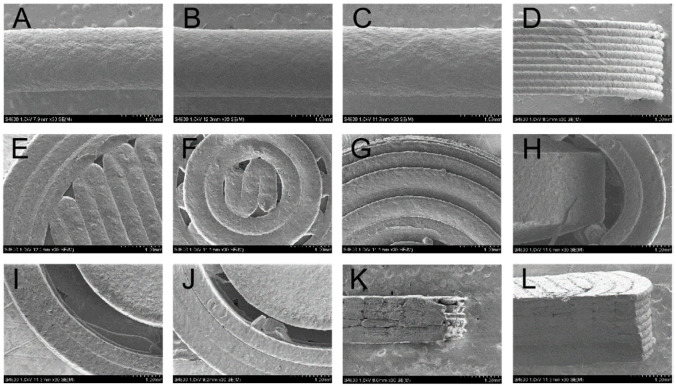
SEM images of the filaments and tablets: (**A**) surface of F90, (**B**) surface of F95, (**C**) surface of F98, (**D**) partial lateral surface of FH-95-266, (**E**) top of FH-95-266, (**F**) and (**G**) top of CH-95-266, (**H**) cross section of FU-95-266, (**I**) cross section of FH-95-266, (**J**) cross section of FH-95-366, (**K**) top of vertical section of FH-95-266, (**L**) bottom of vertical section of FH-95-399. The scale bar represents 1.00 mm.

The SEM images of the filaments and tablets are shown in Figure 5. All the surface of the filaments was smooth, and the smoothest surface was F95 (Figure 5A–C). The diameters of the filaments were uniform. Figure 5D shows the tight and homogenous layer-by-layer deposition of the 3D-printed shells. The filling patterns of the top were different between FH-95-266 (Figure 5E) and CH-95-266 (Figure 5F,G). The flat top of FH-95-266 was filled with the grid format of orthogonal crossing, while the convex top of CH-95-266 was filled with the annular and dense filling pattern. Figure 5H showed the vertical view of cross section of FU-95-266 with the core tablet in the 3D-printed shell. FH-95-266, FH-95-366 and FH-95-399 with the different number of shells (Figure 5I,J; cross section; vertical view) and different layers of top and bottom (Figure 5K,L; vertical section; side view) are shown in Figure 5I–L. It can be seen that the 3D-printed shell was close and seamless, and the layers of bottom in contact with the platform (Figure 5L) were flat and smooth, indicating that the filament melted well before solidification.

The good morphology of the filaments and tablets confirmed the feasibility of using HME and FDM to prepare high resolution core–shell pulsatile tablets.

### 3.4. Weight and Size of Tablets

The weight and size of the tablets are shown in Table 2. The weight of FH-90-266, FH-95-266 and FH-98-266 (tablets with different filament compositions) increased with the increase in HPMC ratio (*p* < 0.05). The weight of FH-95-266, CH-95-266 and FU-95-266 (tablets with different geometric structures) were similar without significant difference (*p* > 0.05), owing to the similar volume of the 3D-printed shells. The weight of FH-95-266, FH-95-366 and FH-95-399 increased naturally with the increase in thickness of the shell (*p* < 0.01).

The variation in tablet weight was considered to be attributed to the following reasons: the slight variation in filament diameter [41,42], the minor damage to the tablets when removed from the platform, wire-drawing during printing, and the small amount of material overflow induced during the stop–start process [32].

As shown in Table 2, the actual height or diameter was similar to the theoretical height or diameter, confirming that the 3D-printed pulsatile tablets could be prepared with high resolution and reproducibility [43].

### 3.5. Hardness of Tablets

The tablets should have sufficient hardness to resist mechanical shocks during transportation and storage [44]. According to the literature [6], FDM 3D printing technology usually provides better mechanical properties compared with other 3D printing technologies.

The hardness of the tablets in three directions are shown in Table 3, which presents directional anisotropy [31]. For all tablets in this study, the hardness measured in direction A was greater than that in direction B or C. The working principle of FDM 3D printing technology was layer-by-layer deposition after the material was fused and extruded [45]. When applying force in direction A (Figure 3), the lower layer supported the upper layer and the overall structure was stable, resulting in larger hardness, whereas the force was applied in direction B or direction C, i.e., if the force was parallel to the deposition plane of layers, slippage and rupture would occur between layers, leading to less hardness of tablets. Moreover, the hardness of all 3D-printed tablets exceeded the maximum detective range of 800 N in direction A, except the hardness of FH-90-266 (468.0 ± 37.42 N). This may be due to the low proportion of HPMC in the filaments, which reduced the hardness. Moreover, the hardness of FH-98-266, FH-95-266 and FH-90-266 gradually decreased in direction B, indicating that when other conditions (geometric structure and thickness of the shell) were same, the hardness of the tablets decreased with the decrease in HPMC proportion (*p* < 0.01). Combined with the hardness results of FH-90-266 in direction A and B, it showed that when the weight ratio of HPMC and PEG 400 was at 90:10, the hardness of the tables might not achieve the desired effect. In addition, the hardness of FH-95-266, FH-95-366 and FH-95-399 gradually increased in direction B, demonstrating that the hardness increased with the increase in the shell thickness (the layers of top/bottom and the number of shells) (*p* < 0.01).

### 3.6. Thermogravimetric Analysis

**Figure 6:**
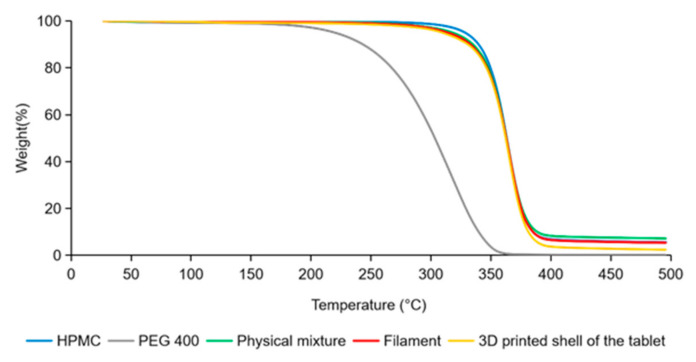
TGA results of HPMC, PEG 400, physical mixture, filament and 3D-printed shell of the tablet.

TGA results of HPMC, PEG 400, physical mixture, filament and the 3D-printed shell of the tablet are shown in Figure 6. The decomposition of HPMC started at 250 °C [46], and the weight loss was less than 1% before the temperature, which indicated HPMC was stable at the HME temperature (140 °C) and FDM 3D printing temperature (200–220 °C). The weight loss (<1%) was supposed due to the evaporation of water according to the literature [16,47]. PEG 400 began to lose weight at about 130 °C and the weight loss was 1% and 5%, respectively, at the temperature of HME and FDM 3D printing, which could be explained by the slow evaporation of PEG 400 [17,48,49]. The profiles of the physical mixture, filament and 3D-printed shell of the tablet were almost overlapped, indicating that the thermogravimetric properties of the samples were consistent with each other. The weight loss of the physical mixture, filament and 3D-printed shell of the tablet started at 240 °C similarly with HPMC, which was considered mainly for the evaporation of water and PEG 400.

Both of HME and FDM 3D printing usually perform at a high temperature, which leads to the degradation of thermally unstable drug and polymer excipients [13]. In this study, drug in the core tablet was intentionally avoided to involve in the HME and FDM 3D printing process, and the excipients (HPMC, PEG 400, physical mixture), the filament and the 3D-printed shell of the tablet were all proven to be thermally stable during the course of HME and FDM 3D printing.

### 3.7. Differential Scanning Calorimeter

**Figure 7:**
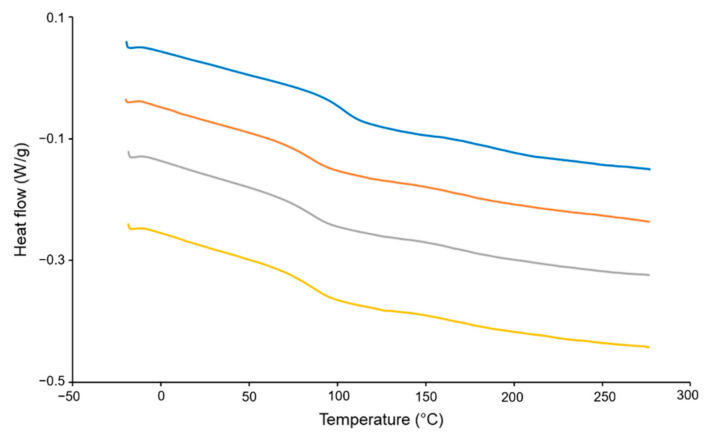
DSC results of HPMC, physical mixture, filament and 3D-printed shell of the tablet.

DSC results of HPMC, the physical mixture, filament and 3D-printed shell of the tablet are shown in Figure 7. The glass transition temperature of HPMC was approximately at 102 °C [46]. For the physical mixture, filament and 3D-printed shell of the tablet, the glass transition temperature was around 85 °C, suggesting that the addition of PEG 400 can effectively reduce the glass transition temperature and heat treatment temperature of HPMC [33,50]. DSC results of the physical mixture, filament and 3D-printed shell of the tablet were similar, indicating that the heating process of HME and FDM 3D printing did not affect the excipients.

### 3.8. X-ray Diffraction

**Figure 8:**
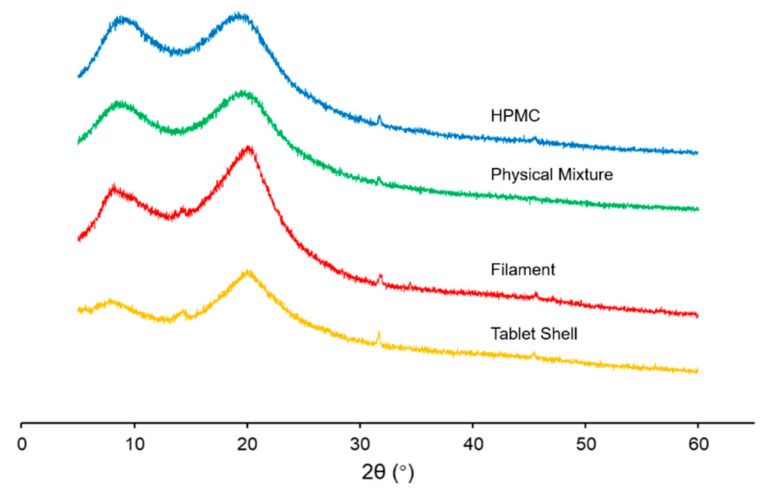
XRD results of HPMC, physical mixture, filament and 3D-printed shell of the tablet.

XRD results of HPMC, the physical mixture, filament and 3D-printed shell of the tablet are shown in Figure 8. The characteristic diffraction peaks of HPMC appeared at 7.92°, 19.98°, 32.80° and 45.60°. Both the positions of the peaks and their corresponding seal intensity were consistent with those reported in literature [51]. The XRD results of the physical mixture were 7.76°, 20.20°, 32.38°, which is similar with HPMC. For the filament and 3D-printed shell of the tablet, a small peak appeared at about 14.25°, which showed that the structure and crystal shape of HPMC had little change after the melting process.

### 3.9. In Vitro Drug Release of Tablets

**Figure 9:**
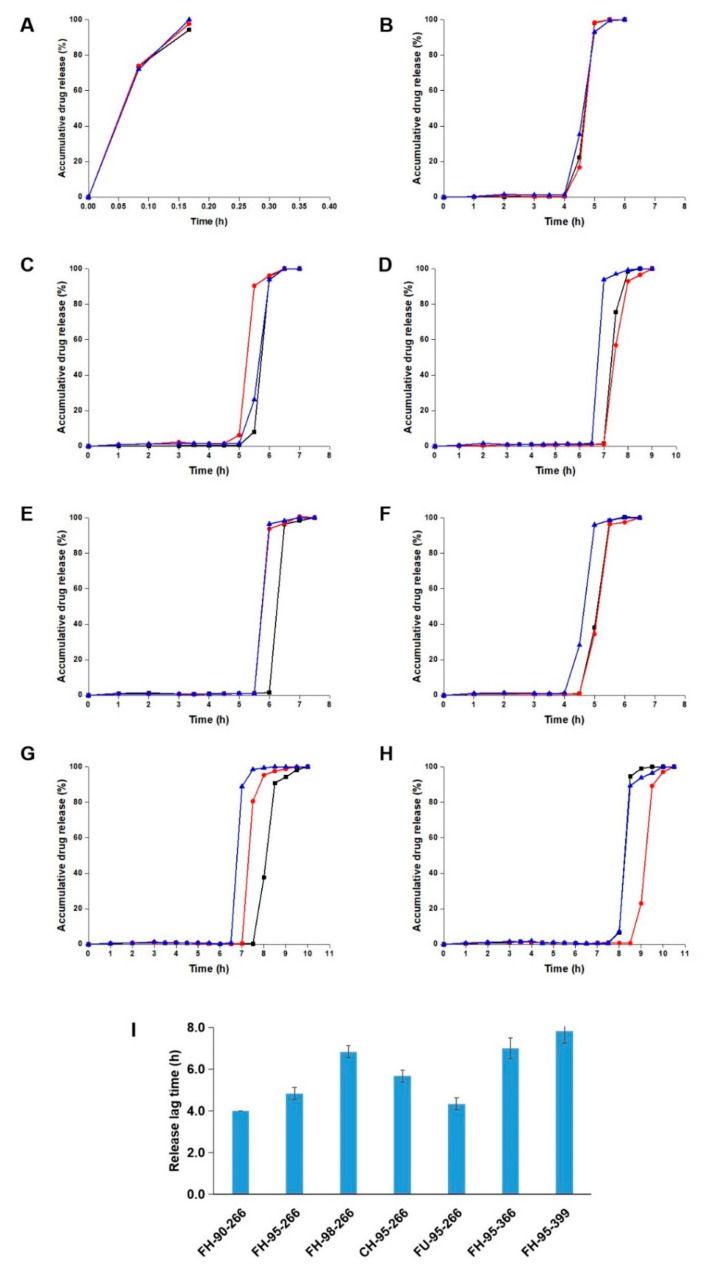
In vitro drug release from (**A**) commercial tablet, (**B**) FH-90-266, (**C**) FH-95-266, (**D**) FH-98-266, (**E**) CH-95-266, (**F**) FU-95-266, (**G**) FH-95-366, (**H**) FH-95-399; (**I**) comparison of the release lag time of the 3D-pinted tablets (*n* = 3).

The drug release curves of the commercial tablet and 3D-printed tablets are shown in Figure 9. The commercial tablet released the drug immediately and the drug was released completely within 10 min. All the printed tablets presented pulsatile drug release behaviour. For the tablets of FH-90-266, FH-95-266, FH-98-266, CH-95-266, FU-95-266, FH-95-366 and FH-95-399, the average time of drug release started from 4.0 h, 4.8 h, 6.8 h, 5.7 h, 4.3 h, 7.0 h and 7.8 h, and finished at 5.0 h, 6.0 h, 8.0 h, 6.0 h, 5.5 h, 8.0 h and 9.5 h, respectively.

By comparing the release behaviour of FH-90-266, FH-95-266 and FH-98-266 (tablets with different filament compositions), it could be found that the tablets with a higher ratio of HPMC had a longer release lag time (4.0 h, 4.8 h and 6.8 h). The reason was considered that the filaments with a higher ratio of HPMC had lower water solubility, which decreased the destruction rate of the shell of the 3D-printed tablet. Comparing the drug release between FH-95-266, CH-95-266 and FU-95-266 (tablets with different geometric structures), the release lag time of FU-95-266 (4.3 h) was observed to be shorter than that of FH-95-266 and CH-95-266 (4.8 h and 5.7 h). This was probably a result of the relatively small joint area between the top/bottom and the shell of FU-95-266, which made the shell easier to be broken in the drug release test. The longer release lag time of CH-95-266 (5.7 h) than that of FH-95-266 (4.8 h) was regarded as its tighter filling pattern of the top. By comparing the drug release of FH-95-266, FH-95-366 and FU-95-399 (tablets with different thicknesses of shell), it could be seen that the tablets with a thicker shell had a longer release lag time (4.8 h, 7.0 h and 7.8 h), as more time was required for water penetrating into the thicker 3D-printed shell and for it being destroyed by erosion after that.

In addition, the drug release rate of the 3D-printed tablets was slower than that of the commercial tablet. This result was considered to be due to the hydrophilic gel matrix material of HPMC in the shell of the 3D-printed tablets, which forms a gel barrier and prolongs drug release as soon as it is exposed to water or gastrointestinal fluid [52].

Consequently, all these 3D-printed pulsatile tablets were proven to achieve drug delayed release in vitro by the design of the core–shell. Personalized lag time of drug release in the range from 4 h to 8 h could be obtained by changing the 3D-printed shell with the filament composition, geometric structure and thickness.

### 3.10. Computed Tomography Imaging

**Figure 10:**
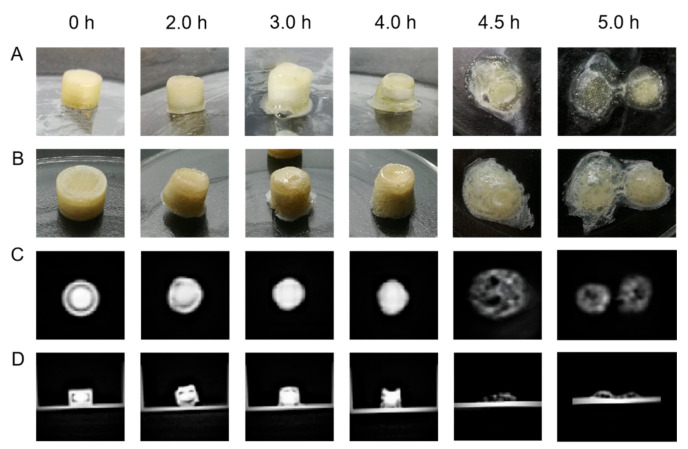
The changes of the 3D-printed tablets during dissolution: pictures of the tablet before (**A**) and after (**B**) freeze-drying process, CT images of cross section (**C**) and vertical section (**D**) of the tablet.

Photographs and CT images of the 3D-printed tablets at different in vitro drug release stages are showed in Figure 10. From the CT images, it can be seen that the core tablet stayed right in the middle of the printed shell and there was a clear gap between them before the dissolution test (0 h). During the dissolution experiment, the 3D-printed shell experienced deformation, thinning and came in contact with the core tablet, attributing to the swelling, eroding and dissolving of HPMC in the shell (at 2.0 h, 3.0 h and 4.0 h). During this period, the core tablet gradually dispersed and spread outward. At 4.5 h, the junction between the top and the side wall of the shell cracked, and the core tablet immediately released. Finally, the shell of the tablet separated into two parts and the core tablet could no longer be observed (at 5 h). The structural change in the 3D-printed tablet at 4.5 h and 5.0 h was highly consistent with the bust release of drug in the dissolution test. Moreover, some of the drug seemed to diffuse into the gel matrix of HPMC (at 4.5 h and 5.0 h), which was supposed to result in a slower release compared with the commercial tablet.

## 4. Conclusions

FDM 3D-printed core–shell pulsatile tablets were fabricated successfully in this study, which were designed with a commercial immediate-release tablet inside and a 3D-printed shell outside. Three factors were considered for the printed shell of the pulsatile tablets, including filament compositions, geometric structures and thicknesses of shell. All the printed tablets showed desired shapes and elegant appearances, and the factors were found to affect the weight, size, hardness and in vitro release of the tablets. The components of the filaments were proved to be stable during the HME and 3D printing process by TGA, DSC and XRD measurement. The tablets achieved personalized lag time ranging from 4 h to 8 h in the drug release test in PBS (pH 6.8). The CT images revealed that the destruction of the structure of the printed shell led to the release of the drug in the core tablet. This study explored a novel method to prepare personalized oral pulsatile tablets combining the advantage of FDM 3D printing with traditional pharmaceutical technology, which avoided heat treatment to the drug. The developed core–shell pulsatile tablets showed a potential platform for personalized administration in the treatment of circadian rhythm diseases.

## Figures and Tables

**Table 1:** Design of the outer shell of the core–shell 3D-printed pulsatile tablets.

Tablet	Filament Composition	Geometric Structure		Number of Shells/Layers		
		Shape of the Top	Direction of the Core Tablet	Shells	Top Layers	Bottom Layers
FH-90-266	F90	flat	horizontal	2	6	6
FH-95-266	F95	flat	horizontal	2	6	6
FH-98-266	F98	flat	horizontal	2	6	6
CH-95-266	F95	convex	horizontal	2	6	6
FU-95-266	F95	flat	upright	2	6	6
FH-95-366	F95	flat	horizontal	3	6	6
FH-95-399	F95	flat	horizontal	3	9	9

The thickness of each shell and layer was 0.4 mm and 0.2 mm, respectively.

**Table 2:** Weight and size of the core–shell 3D-printed tablets (mean ± SD, *n* = 5).

Tablet	Weight/mg	a/mm	a*/mm	b/mm	b*/mm	c/mm	c*/mm
FH-90-266	328.5 ± 4.2	5.80	5.89 ± 0.02	9.20	9.13 ± 0.03	-	-
FH-95-266	350.0 ± 7.3	5.80	5.87 ± 0.04	9.20	9.17 ± 0.05	-	-
FH-98-266	369.8 ± 9.4	5.80	5.93 ± 0.03	9.20	9.25 ± 0.03	-	-
CH-95-266	342.1 ± 7.3	5.80	5.92 ± 0.03	9.20	9.22 ± 0.08	-	-
FU-95-266	347.4 ± 14.0	8.80	8.88 ± 0.05	9.20	9.21 ± 0.05	6.20	6.18 ± 0.05
FH-95-366	442.9 ± 6.5	5.80	5.88 ± 0.03	10.00	10.04 ± 0.03	-	-
FH-95-399	528.9 ± 11.9	7.00	7.11 ± 0.04	10.00	9.97 ± 0.06	-	-

a, b and c represented the theoretical value in Figure 1, a*, b* and c* represented the corresponding actual value, respectively.

**Table 3:** Hardness of the core–shell 3D-printed tablets (mean ± SD) (*n* = 6).

Tablet	Direction A	Direction B	Direction C
FH-90-266	468.0 ± 37.42	127.5 ± 16.50	/
FH-95-266	>800	319.4 ± 28.31	/
FH-98-266	>800	404.2 ± 19.15	/
CH-95-266	>800	198.0 ± 38.99	/
FU-95-266	>800	301.5 ± 29.03	358.3 ± 35.16
FH-95-366	>800	338.8 ± 24.84	/
FH-95-399	>800	488.8 ± 39.64	/

## Data Availability

Not applicable.
